# Competitive Traits Are More Important than Stress-Tolerance Traits in a Cadmium-Contaminated Rhizosphere: A Role for Trait Theory in Microbial Ecology

**DOI:** 10.3389/fmicb.2018.00121

**Published:** 2018-02-12

**Authors:** Jennifer L. Wood, Caixian Tang, Ashley E. Franks

**Affiliations:** ^1^Department of Physiology, Anatomy and Microbiology, La Trobe University, Melbourne, VIC, Australia; ^2^Center for AgriBiosciences, La Trobe University, Melbourne, VIC, Australia

**Keywords:** cadmium, rhizosphere, community assembly, CSR theory, functional traits

## Abstract

Understanding how biotic and abiotic factors govern the assembly of rhizosphere-microbial communities is a long-standing goal in microbial ecology. In phytoremediation research, where plants are used to remediate heavy metal-contaminated soils, a deeper understanding of rhizosphere-microbial ecology is needed to fully exploit the potential of microbial-assisted phytoremediation. This study investigated whether Grime's competitor/stress-tolerator/ruderal (CSR) theory could be used to describe the impact of cadmium (Cd) and the presence of a Cd-accumulating plant, *Carpobrotus rossii* (Haw.) Schwantes, on the assembly of soil-bacterial communities using Illumina 16S rRNA profiling and the predictive metagenomic-profiling program, PICRUSt. Using predictions based on CSR theory, we hypothesized that Cd and the presence of a rhizosphere would affect community assembly. We predicted that the additional resource availability in the rhizosphere would enrich for competitive life strategists, while the presence of Cd would select for stress-tolerators. Traits identified as competitive followed CSR predictions, discriminating between rhizosphere and bulk-soil communities whilst stress-tolerance traits increased with Cd dose, but only in bulk-soil communities. These findings suggest that a bacterium's competitive attributes are critical to its ability to occupy and proliferate in a Cd-contaminated rhizosphere. Ruderal traits, which relate to community re-colonization potential, were synergistically decreased by the presence of the rhizosphere and Cd dose. Taken together this microcosm study suggests that the CSR theory is broadly applicable to microbial communities. Further work toward developing a simplified and robust strategy for microbial CSR classification will provide an ecologically meaningful framework to interpret community-level changes across a range of biomes.

## Introduction

Understanding how rhizosphere-microbial communities assemble and are maintained temporally is a major goal in microbial ecology that is relevant to a wide range of disciplines including: plant-pathogen interactions (Pieterse et al., 2014), agriculture (Welbaum et al., 2004), community ecology (Fitzsimons and Miller, 2010), microbiome research (Costello et al., 2012), and phytoremediation (Thijs et al., 2016). In the field of phytoremediation, where plants are utilized to remove contaminants from soils, manipulation of the rhizosphere community via the addition of beneficial microorganisms has been used to improve remediation rates (De Souza et al., 1999; Ma et al., 2009; Liu et al., 2015; Wood et al., 2016a). However, to fully exploit this potential, a greater understanding of factors driving community assembly in the rhizosphere during phytoremediation are needed.

At a local scale, the assembly of rhizosphere communities is generally accepted to be under the influence of biotic factors predominant amongst which is the plant itself (Grayston et al., 1998; Smalla et al., 2001; Aira et al., 2010). Multiple abiotic factors also play a role in shaping the community, including temperature (Melent'ev et al., 2000); soil clay content (Latour et al., 1999); soil moisture and pH (Nuccio et al., 2016). Rhizosphere communities associated with phytoremediation technologies are additionally shaped by contaminants present in the soil. For instance, in the presence of soil contaminants such as hydrocarbons or heavy metals, plant rhizospheres have been shown to contain an enrichment of hydrocarbon-degrading (Siciliano et al., 2001; Yergeau et al., 2014) or heavy-metal-resistant (Mengoni et al., 2001) microorganisms, respectively.

Despite the accumulating information surrounding factors that influence rhizosphere-community assembly, the field is limited in its ability to predict how communities assemble or the ability of an isolate to infiltrate a rhizosphere community. The development of broad ecological frameworks for the classification and interpretation of microbial communities is considered a priority for the advancement of microbial ecology and may provide a strategy for understanding community assembly (Prosser, 2015; Widder et al., 2016). Emerging trends such as the use of community aggregated traits, which utilize functional information derived from a community metagenome, provide opportunities to describe microbial communities in terms of ecologically relevant functional traits (Fierer et al., 2014). In ocean communities, metagenomic approaches have been used to show a separation in microbial-community functional traits between subsurface-phototropic zones in the water column and near-ocean-floor depths (DeLong et al., 2006). Traits enriched close to the surface were related to high rates of carbon capture (high rates of metabolic activity and foraging abilities) whilst traits related to persistence in unproductive environments (such as polysaccharide production) were enriched in deep waters (DeLong et al., 2006).

For highly diverse microbial communities, such as soil-microbial communities, construction of a metagenome that adequately samples community diversity requires large volumes of sequencing data which can be prohibitive for many researchers (Howe et al., 2014). However, the relative accessibility of 16S rRNA profiling, the advent of predictive metagenomic profiling and growing reference-genome databases, present an opportunity for multiple-trait-based investigations of microbial communities using 16S rRNA datasets (Langille et al., 2013).

The use of functional traits (either at the species or the community level) to understand processes that govern the assembly of communities is a major goal of the competitor/stress-tolerator/ruderal (CSR) theory (Grime and Pierce, 2012). The CSR theory proposes that organisms face a three-way resource trade-off between the investment in traits that facilitate: competition with neighbors for resources (Competitive traits); survival in underproductive environments (Stress-tolerant traits); and survival in highly disturbed environments (Ruderal traits) (Grime, 1974, 1977; Grime and Pierce, 2012). Using functional traits, an organism can be ascribed a C, S, or R classification, with intermediate (e.g., CS, CR, CSR etc.) classifications also recognized (Hodgson et al., 1999). Levels of environmental stress, disturbance and competition collectively govern which class of CSR traits can persist in a community, thus providing a link between community function and environmental constraints.

Although the CSR theory was originally developed to explain plant-community assembly, its principles are generalizable and have been recognized as being theoretically applicable to microbial communities (Prosser et al., 2007; Krause et al., 2014; Thijs et al., 2016). A number of trait trade-offs akin to those proposed in the CSR theory have been described in microorganisms (Lipson et al., 2009; Wallenstein and Hall, 2012; Litchman et al., 2015), including trade-offs between stress tolerance and competitive ability which are likely to affect community assembly in contaminated rhizospheres (Ferenci and Spira, 2007).

The major aim of this study was to determine the applicability of trait-based theory in explaining the impact of cadmium (Cd), and the presence of a Cd-accumulating plant with phytoremediation potential, *Carpobrotus rossii* (Haw.) Schwantes (Zhang et al., 2014), on the assembly of rhizosphere-bacterial communities using 16S rRNA profiles. We access community-level functional-traits using predictive metagenomics profiling, which were allocated C, S, or R classifications based on the theory underlying the CSR hypothesis. We hypothesized that both selective pressures would alter community composition, with the additional resource availability in the rhizosphere enriching for competitive life strategists and the presence of Cd selecting for stress-tolerators.

## Materials and methods

### Plant growth experiment

Plant growth experiments were conducted in glasshouses at La Trobe University, Bundoora, Victoria, Australia, using the Australian native succulent *Carpobrotus rossii*, which has an ability to accumulate Cd (Zhang et al., 2014). Soil used in this experiment was a sandy loam collected from the topsoil (0–25 cm) of the La Trobe University Agricultural Reserve, Bundoora, Victoria, Australia. Basic soil properties have been described previously in Wood et al. (2016b). Prior to the experiment, soil was passed through a 2-mm sieve and air-dried.

Experimental procedures are described in detail in Wood et al. (2016b), briefly: Soil (0.25 kg) was spiked with CdCl_2_ solutions to achieve 20 or 100 mg kg^−1^ CdCl_2_. Distilled H_2_O was used as a negative control. Basal nutrients were added to each bag of soil to achieve the following concentrations (mg kg^−1^ soil): 150 K_2_SO_4_, 150 KH_2_PO_4_, 90 CaCl_2_·2H_2_O, 21 MgSO_4_·7H_2_O, 14 MnCl_2_, 1.081 CuCl_2_·H_2_O, 10.33 ZnSO_4_·7H_2_O, 0.67 H_3_BO_3_, and 0.15 Na_2_MoO_4_·2H_2_O. Sterile distilled H_2_O was then added to wet the soil to 80% field capacity.

After 2 weeks of incubation, soil was transferred to washed forestry tubes and *Carpobrotus rossii* plantlets were transplanted into half of the pots. Pots were maintained at 80% field capacity, using sterile distilled H_2_O, for 8 weeks.

Bulk-soil samples were collected from replicates without plants by using 50 ml centrifuge tubes to take 3-cm cores to a depth of 5-cm. Cores were stored at −80°C until DNA extraction. Samples of rhizosphere soil were collected from replicates containing plants by gently removing plants from their pots and shaking away loose soil from the roots. The rootstocks were stored at −80°C until DNA was extracted. At the time of DNA extraction, soil left clinging to the roots was used to extract rhizosphere community DNA.

### Soil DNA extraction and sequencing

Community gDNA was extracted from bulk and rhizosphere soil (0.25 g) using a power-soil DNA isolation kit (MOBIO) as per manufacturer's instructions. DNA concentrations were recorded using an Implen P-class Nanophotometer (p-330). All samples were normalized to working concentrations of 5 ng μl^−1^ and stored at −20°C until required.

Libraries were prepared for sequencing on the Illumina Miseq following the Illumina “16S Metagenomic Sequencing Library Preparation” protocol (Illumina, Part # 15044223 Rev. B). Locus specific primers used were the universal 16S rRNA primers S-D-Bact-0341-b-S-17 (5′-CCTACGGGNGGCWGCAG) and S-D-Bact-0785-a-A-21 (5′-GACTACHVGGGTATCTAATCC) which target the V3-V4 region of the bacterial 16S rRNA gene. Primers had forward (5′-TCGTCGGCAGCGTCAGATGTGTATAAGAGACAG) and reverse (5′-GTCTCGTGGGCTCGGAGATGTGTATAAGAGACAG) Illumina overhang adaptors merged to the 5′ ends.

PCRs were performed in 25 μl reactions using: 5 nM of each forward and reverse primer, 2 × KAPA HiFi HotStart ReadyMix and 12.5 ng of genomic DNA template. PCR cycle settings for the amplification of the bacterial V3-V4 region were as follows: 95°C denaturation for 3 min, followed by 25 thermal cycles of 30 s at 95°C, 30 s at 55°C, and 30 s at 72°C, followed by an extension step at 72°C for 10 min. To normalize libraries prior to pooling, the DNA content of PCR reactions were quantified fluorimetrically using a Qubit Flourometer (Invitrogen, CA, USA). Prepared libraries were spiked with 20% Phi-X prior to paired-end sequencing (2 × 250) on an Illumina MiSeq platform.

### Bioinformatic analysis

Raw, demultipexed, fastq files were re-barcoded, joined and quality-filtered using UPARSE OTU clustering pipeline (Edgar, 2013). Joined paired-end reads were quality filtered by discarding reads with total expected errors > 1.0 and singletons were removed from the dataset. Reads which could not be assembled were discarded.

Operational taxonomic units (OTUs) were clustered with a 97% similarity cutoff using UPARSE clustering algorithm (USEARCH version 8.1.1861 http://drive5.com/uparse/). Taxonomic assignments were performed using the USEARCH UTAX algorithm. Reference databases were created using the RDP_trainset_15 dataset, available from the UTAX downloads page (http://drive5.com/usearch/manual/utax_downloads.html). The minimum percentage identity required for an OTU to consider a database match a hit was 90%. OTUs identified as chloroplasts and mitochondrial DNA were removed from the data. Raw fastq files for this project have been deposited with the NCBI SRA database and can be accessed using Bioproject ID: PRJNA384274 or SRA study ID: SRP105232.

#### Generation of predicted metagenomic profiles using PICRUST

The functional potential of bacterial communities was estimated using Phylogenetic Investigation of Communities by Reconstruction of Unobserved States (PICRUSt) (Langille et al., 2013). Functional annotations were assigned using the Kyoto encyclopedia of genes and genomes (KEGG) database to generate sample × functional counts tables (Kanehisa and Goto, 2000).

In accordance with the PICRUSt predictive metagenomic pipeline, taxonomic assignments were performed using Qiime closed reference OTU picking against the GreenGenes gg_13_5 reference set (Caporaso et al., 2010). The minimum percentage identity required for an OTU to consider a database match a hit was 97%. Prior to predicting the metagenome, sample × OTU-count data was normalized by 16S rRNA copy-number using the normalize_by_copy_number.py script. This pipeline resulted in a sample × KEGG ortholog (KO)-count table which was used to determine functional trait enrichment. KOs represent cross-species genome annotations whereby experimentally derived gene-function relationships from one genome are extended to orthologous genes in all available genomes.

### Statistical analysis

Alpha- and beta-diversity analyses were performed on OTU tables rarefied to a depth of 3500 reads using the “phyloseq” and “vegan” packages in the R programming language (version 3.2.2) (Dixon, 2003; McMurdie and Holmes, 2013; R Development Core Team, 2014). One rhizosphere sample (0 mg Cd kg^−1^ soil) that contained significantly <3,500 reads was discarded.

Two-way ANOVAs were performed to test for differences in OTU richness and Shannon diversity via the “aov” function. Normality and variance homogeneity of the data were tested using the “shapiro.test” and “bartlett.test” functions. *Post-hoc* testing of significance was performed using Tukey's honest significant difference (HSD) test. Community beta-diversity was examined using the UniFrac distance metric and NMDS ordinations (Lozupone and Knight, 2005). Differences in community structure were tested for via two-way PEMANOVAs using the “Adonis” function available in R “vegan” package (Dixon, 2003).

Differential abundance testing of OTUs was performed in R (version 3.3.1) using the DESeq2 extension available within the “phyloseq” package (McMurdie and Holmes, 2013; Love et al., 2014). Tests were performed by applying model-fitting normalization to unrarefied OTU tables as recommended by McMurdie and Holmes (2014).

### Identification of predicted enriched community aggregated traits

The enrichment of functional pathways was calculated in a pair-wise fashion by comparing each treatment group to the un-contaminated bulk-soil samples. KOs that were over represented in treatment groups for each pair-wise comparison were identified using differential expression analysis based on the negative binomial distribution using the R package DESeq2 and phyloseq (Love et al., 2014). Enriched KOs were mapped to their associated functional pathways using custom R scripts and the KEGG Ontology database (Release 79.1). KEGG pathways can contain as few as 1 KO and individual KOs can be represented in multiple KEGG pathways. Thus, we employed a bootstrapping method, similar to that described in DeLong et al. (2006), to identify KEGG pathways with statistically more enriched-KO hits than expected under a null model (i.e., the enriched functional pathways).

For each pair-wise comparison, the difference between observed and expected instances of enriched KOs (“KO hits”), for each given KEGG pathway, were calculated for both treatment groups. The expected number of KO hits per pathway were calculated by drawing a list of random KOs (n), without replacement, from the total list of KO identifiers present in the collective predicted functional genome. Where n = number of unique KO identifiers enriched in a given treatment group. This was repeated 1,000 times, and the median differences calculated.

To identify those median differences that were statistically unlikely to have occurred by chance, this process was repeated for each treatment group, except the list of KO identifiers (n) were sampled at random for observed *and* expected categories. Again, 1,000 repeat calculations were performed, and the data organized from least difference to most difference. The confidence intervals were provided by the appropriate percentile differences, that is for 95% confidence intervals the 5% limit was provided by the 50th difference and the 95% limit was provided by the 950th difference from the ordered list. If the difference of medians was above the 95% limits, the KEGG pathway was considered be significantly enriched.

To identify any functional pathways that were significantly under-represented in the predicted metagenomes (i.e., pathways hits that were likely to be due to KO redundancy), the above bootstrapping procedure was employed using the predicted metagenome as the source for the “observed” and the total list of KOs mapped to their respective reference pathways as the “expected” data. Any functional pathways that fell below the 5% limit (i.e., were significantly underrepresent in the predicted functional profile of our data) were excluded for the final analysis.

Manual inspection of the enriched pathways against the KEGG database were performed to further manually cross check for redundancy and to categorize functional pathways as competitive (C), stress tolerant (S) or ruderal (R) functional traits using criteria outlined briefly below (Table 1) and expanded upon in the Supplementary Material (Tables S1–S5).

**Table 1:** Definitions used to define microbial traits as competitive, stress-tolerant or ruderal, adapted from Grime and Pierce (2012).

**Trait definition**	**Potential modes for microorganisms**	**Macro (plant community) example**	**Micro example**
Competitive trait: traits constituting an investment in the monopolization of local resources	Local resource monopolization via increased capacity to capture resources	Large leaves; high chlorophyll concentration; large canopy spread	Increase in membrane transporters; Siderophore monopolization of iron
	Local resource monopolization via direct inhibition of neighbors	Allelochemical production; shading	Increased antibiotic production; biofilm formation
Stress-tolerant trait: traits that facilitate survival in chronically underproductive environments (i.e., traits improving resource conservation)	Prevention of resource loss due to damage	Mechanical defenses such as spines; chemical deterrents of herbivory	Increase in osmoregulation capacity; ability to alter membrane fluidity; UV absorption via melanin or pigments
	Mitigation of cellular damage	Detoxification mechanisms; production of free-radicle scavenges	Increase in DNA repair pathways; production of free-radicle scavenges
Ruderal trait: traits constituting an investment in processes that permit the re-establishment of a population	Rapid re-establishment via increase in growth-limiting metabolic processes	High photosynthetic capacity	Increased capacity for central metabolic flux
	Rapid re-establishment via increase in reproduction-limiting structures	High seed number; short life cycle	Increase potential for ribosome production or nucleotide production

To visualize changes in functional traits we constructed a 3-dimensional representation depicting the net change in functional traits for each microcosm treatment relative to the base-line soils. Each trait-axis was scaled by the number of traits detected for each CSR class respectively. Scores for each CSR category, within each microcosm were calculated using the following equation:

$$\begin{array}{l} \left[\left(\frac{no.\;traits\;enriched\;in\;microcosom}{total\;functional\;traits}\right)\right]\\ \;-\left(\frac{no.\;traits\;enriched\;in\;baseline}{total\;functional\;traits}\right)]\times 100 \end{array}$$

## Results

**Figure 1:**
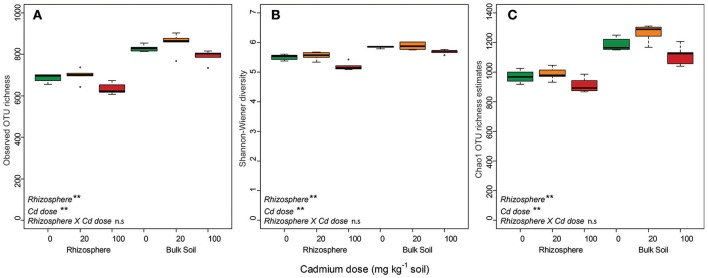
Box plots of observed OTU richness **(A)**, Shannon-Wiener diversity **(B)** and Chao1 OTU richness estimates **(C)** for bacterial communities across treatments. Color is indicative of Cd treatment: Green = 0 mg Cd kg^−1^ soil; Amber = 20 mg Cd kg^−1^ soil; Red = 100 mg Cd kg^−1^ soil. Results of two-way ANOVAs are reported in the bottom left of panels: ^**^p < 0.01; n.s, not significant at *p* ≤ 0.05. Whiskers extend to a maximum of 1.5 × IQR beyond the box. *n* = 5.

Controlled microcosm studies were used to disentangle the impact of Cd and the presence of a Cd-accumulating plant rhizosphere on bacterial community assembly via the examination of operational taxonomic units (OTUs). Changes in OTU richness and Shannon-Wiener diversity were used to examine the extent to which each selective pressure filtered OTUs from the environment, whilst changes in taxonomic structure were used to examine the extent to which each selective pressure altered community competition dynamics. The observed and Chao1-estimated OTU richness and Shannon-Wiener diversity indices of bacterial communities associated with the *Carpobrotus rossii* were significantly lower when compared to bulk-soil communities (Figure 1). *Post-hoc* testing revealed that the 100 mg kg^−1^ Cd treatment significantly reduced OTU richness and diversity compared to the 0 and 20 mg kg^−1^ Cd treatments (*p* < 0.05). There was no significant difference in OTU richness or diversity between the 0 and 20 mg kg^−1^ Cd treatments.

**Figure 2:**
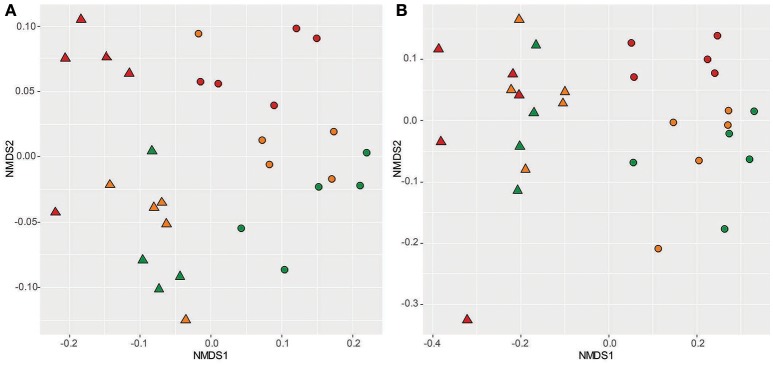
Influence of Cd and the plant rhizosphere on bacterial community structure. Non-metric multidimensional scaling ordination of bacterial communities using weighted **(A)** and unweighted **(B)** UniFrac distance. Ordination stress values are 0.10 and 0.12, respectively. “°” indicate bulk-soil community samples, “Δ” indicate rhizosphere community samples. Color is indicative of Cd treatment: Green = 0 mg Cd kg^−1^ soil; Amber = 20 mg Cd kg^−1^ soil; Red = 100 mg Cd kg^−1^ soil.

We observed significant differences in the taxonomic structure of rhizosphere and bulk-soil communities using both weighted (Figure 2A; *R* = 0.76, *p* < 0.05) and unweighted (i.e., binary transformed) Unifrac distances (Figure 2B; *R* = 0.85, *p* < 0.05). Interestingly, the grouping of communities due to Cd dose was stronger in the rhizosphere than in bulk soils for weighted Unifrac ordinations (Figure 2A: R_rhizosphere_ = 0.48, *p* < 0.01; R_bulk soil_ = 0.31, *p* < 0.05) but was diminished to a point where significant differences could no longer be detected when data were binary transformed (Figure 2B; R_rhizosphere_ = n.s; R_bulk soil_ = 0.31, *p* < 0.01). The importance of abundance information in distinguishing treatments due to Cd dose in the rhizosphere indicates that Cd affected the distribution of OTU abundances within these communities more than taxonomic composition.

### Trait-based analysis of microbial communities

Predictive metagenomics software (PICRUSt) was used as a basis for a trait-based examination of communities to test whether a macro-ecological trait-based theory (CSR theory) could be used to explain the impact of Cd and the plant rhizosphere on community assembly in terms of stress, competition and disturbance. By mapping significantly enriched KOs to KEGG functional pathways, we identified 28 and 19 functional pathways that were enriched in rhizosphere and bulk-soil communities, respectively (Table 2), and 15 and 17 functional pathways that were enriched in uncontaminated and highly contaminated soils, respectively (Table 2).

**Table 2:** Functional pathways identified as relating to competition (C), stress-tolerance (S) or ruderal life strategies (R), and their patterns of enrichment for each microcosm relative to base-line communities from no-Cd bulk-soil treatments.

**Cd dose**	**Competitive traits**		**Stress tolerant traits**		**Ruderal traits**	
	**Enriched**	**Decreased**	**Enriched**	**Decreased**	**Enriched**	**Decreased**
**BULK-SOIL COMMUNITIES**						
20 mg	–	–	–	–	–	Carbon metabolism
100 mg	–	–	Porphyrin and chlorophyll metabolism	–	–	Biosynthesis of amino acids; Carbon-fixation pathways in prokaryotes; TCA cycle; Oxidative phosphorylation
**RHIZOSPHERE COMMUNITIES**						
0 mg	ABC transporters; Biosynthesis of type II polyketide backbone; Clavulanic acid biosynthesis	Carbapenem biosynthesis	Proteasome; Ascorbate and aldarate metabolism	Mismatch repair	–	2-Oxocarboxylic acid metabolism; Aminoacyl-tRNA biosynthesis; Biosynthesis of amino acids; Pyrimidine metabolism; Ribosome; One carbon pool by folate
20 mg	ABC transporters; Biosynthesis of siderophore group nonribosomal peptides; Clavulanic acid biosynthesis	Acarbose and validamycin biosynthesis; Polyketide sugar unit biosynthesis	Ascorbate and aldarate metabolism; Proteasome	Mismatch repair; Thiamine metabolism‘	–	2-Oxocarboxylic acid metabolism; Aminoacyl-tRNA biosynthesis; Biosynthesis of amino acids; Ribosome
100 mg	Biosynthesis of type II polyketide products; Clavulanic acid biosynthesis; Staurosporine biosynthesis	Acarbose and validamycin biosynthesis; Polyketide sugar unit biosynthesis	ABC transporters;	–	–	2-Oxocarboxylic acid metabolism; Aminoacyl-tRNA biosynthesis; Biosynthesis of amino acids; Carbon-fixation pathways in prokaryotes; Oxidative phosphorylation; Pyrimidine metabolism; Ribosome

*See Tables S1–S3 for explicit rationalizations, with reference to supporting literature, as to why each microbial trait was classified as either a C, S, or R trait*.

A total of nine functional traits were classified as relating to the monopolization of local resources (C traits), six were classified as processes that maintain cell integrity (S traits) and nine were classified as relating to the ability of populations to re-establish in circumstances of frequent disturbance (R traits). One trait (ABC transporters) was conditionally classified as either C or S depending on the nature of the enriched transporters (Tables S1, S2). A suite of six unclassified functional pathways were related to foraging whilst others were hypothesized to reflect difference in resource type between microcosm treatments (Tables S4, S5).

**Figure 3:**
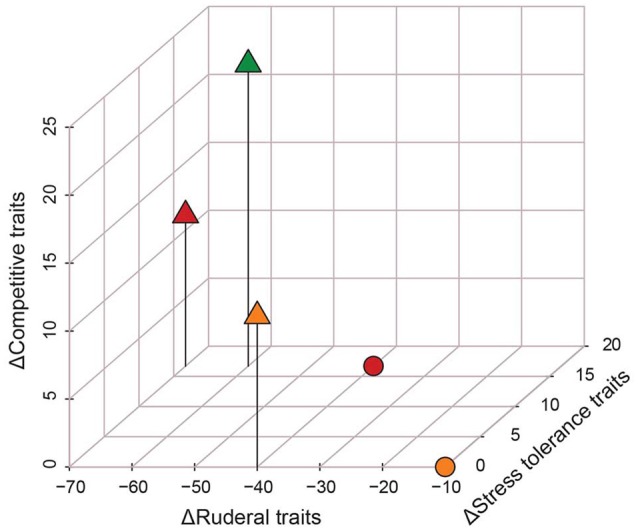
Three-dimensional representation of change (Δ) in the number of functional traits for each microcosm treatment relative to the base-line soils (i.e., no-Cd bulk soils). Axis have been scaled by the number of traits detected for each CSR class. “°” indicate bulk-soil community samples, “Δ” indicate rhizosphere community samples. Color is indicative of Cd treatment: Green = 0 mg Cd kg^−1^ soil; Amber = 20 mg Cd kg^−1^ soil; Red = 100 mg Cd kg^−1^ soil.

Classification of the predicted enriched pathways into functional traits revealed distinct traits associated with each environment (Table 2, Figure 3). When compared to base-line soils (uncontaminated bulk soil), all rhizosphere treatments scored high on the C axis and low on the R axis (Figure 3). In the presence of Cd, C-scores for rhizosphere treatments were reduced due to the enrichment of competitive traits (Acarbose and validamycin biosynthesis and polyketide sugar unit biosynthesis) in the base-line soil (Figure 3, Table 2). Bulk soils treated with 20 mg Cd kg^−1^ were the closest to base-line soils. As Cd dose increased, there was a decrease in R scores within bulk and rhizosphere communities.

### Impact of rhizosphere and cadmium on OTU abundances

Six hundred and three OTUs exhibited a significant change in abundance in response to the presence or absence of the rhizosphere, whilst 149 OTUs exhibited a significant change in abundance in response Cd dose when comparing controls to the 100 mg Cd kg^−1^ soil treatment. In contrast, only 6 OTUs exhibited a significant change in abundance in response Cd dose when comparing controls to the 20 mg Cd kg^−1^ treatment (data not shown).

**Figure 4:**
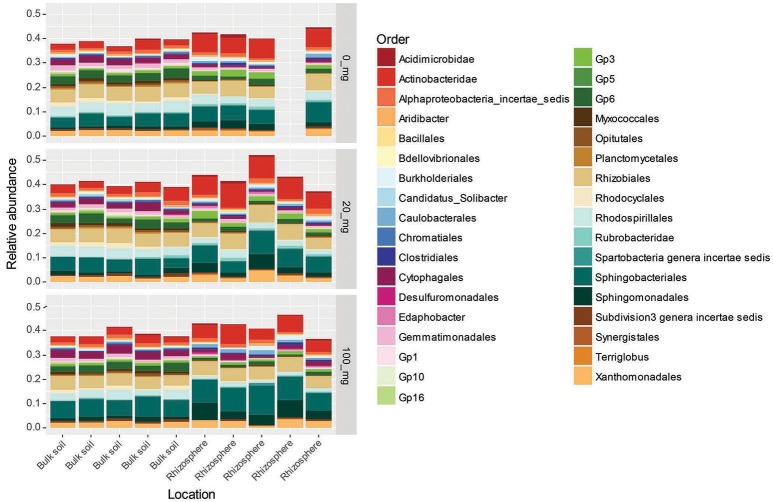
Relative abundance of OTUs found to respond significantly (*p* < 0.05) to the presence of rhizosphere at 0, 20, and 100 mg Cd kg^−1^ soil. OTUs are clustered by order. Only orders that represented >0.5% of the total community are represented. Absent bar represents replicate that was excluded due to under sampling. Orders denoted as Gp “X” are groups belonging to the phylum Acidobacteria.

The major phyla increasing in response to the presence of the rhizosphere were the Actinobacteridae (Actinobacteria), Sphingobacterales (Bacteroidetes) and Sphingomonadales (alpha-Proteobacteria) (Figure 4). Interestingly, the increase in abundance observed for the Sphingobacterales and Sphingomonadales was only apparent in the presence of Cd. The order Rhizobiales were another major group responding to the presence/absence of the rhizosphere. However, at the level of order, there was no clear trends in abundance changes, suggesting that there was a turnover in OTUs (i.e., some decreasing whilst others increased). The major Orders that decreased in abundance due to the presence of the rhizosphere were the Cytophagales (Bacteroidetes) and Acidobacteria order Gp6 (Figure 4).

**Figure 5:**
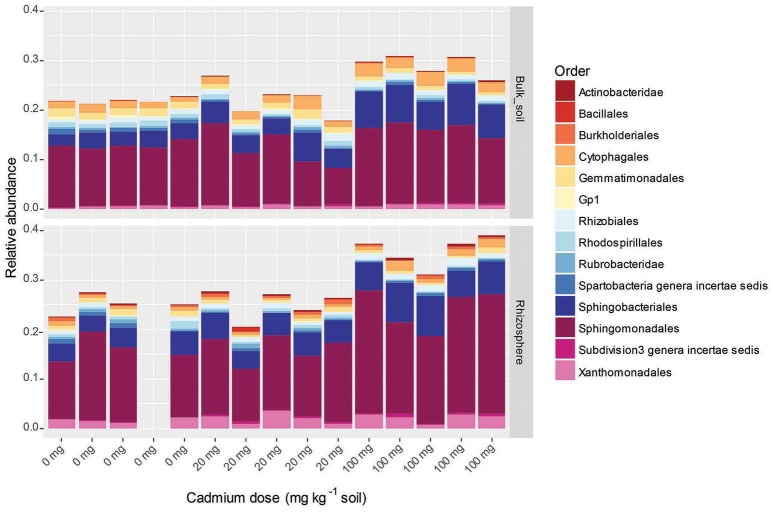
Relative abundance of OTUs found to respond significantly (*p* < 0.05) to Cd dose in the rhizosphere and bulk soils. OTUs are clustered by order. Only orders that represented >0.5% of the total community are represented. Absent bar represents replicate that was excluded due to under sampling. Orders denoted as Gp “X” are groups belonging to the phylum Acidobacteria.

The three major taxonomic Orders represented by OTUs responding to Cd dose were the Sphingomonadales (phylum alpha-Proteobacteria), Sphingobacterales (phylum Bacteroidetes) and Cytophagales (phylum Bacteroidetes). The Cd-induced increase in abundance for all three Orders was only apparent in the 100 mg Cd kg^−1^ treatment (Figure 5).

## Discussion

Trait-based ecology rests on the hypothesis that physical and physiological traits of an organism reflect adaptations to the environmental conditions an organism has been exposed to over evolutionary time. In particular, Grimes CSR theory relates traits classified as Competitive (C), Ruderal (R) or Stress tolerant (S) to the levels of environmental disturbance and stress (Grime and Pierce, 2012). This study aimed to determine the applicability of CSR theory, which is derived from macro-community ecology, in developing an understanding of factors governing bacterial-community assembly in the rhizosphere of a Cd-accumulating plant using controlled microcosms. Both the presence of the rhizosphere and Cd altered bacterial-community structure and bacterial traits classified as relating to competitive, stress-tolerant or ruderal life-strategies broadly followed predictions made by the CSR theory. We found that competitive traits were effective at discriminating between rhizosphere and bulk-soil microcosms, whilst stress tolerance traits were enriched with Cd dose, but only in bulk-soil communities.

### Competitive traits are enriched in the rhizosphere

Grimes CSR theory predicts that in undisturbed environments where resources are not limiting competitive life strategies will prevail (Grime and Pierce, 2012). Due to the excess resource availability in the rhizosphere, in the form of root exudates, we hypothesized that the plant would filter for competitive life-strategists (Hartmann et al., 2008; Lugtenberg and Kamilova, 2009). Our data support this hypothesis as, relative to base-line soils, rhizosphere communities scored higher on the C axis due to an enrichment of traits linked to microbial competition for resources including the production of siderophores and antimicrobial compounds (Figure 3, Table 2). Reductions in the Shannon-Weiner diversity and OTU richness of rhizosphere communities compared to bulk-soil communities suggest that the rhizosphere was filtering OTUs from the wider bulk-soil community (Figure 1). That the rhizosphere can act as an ecological filter for community assembly is supported by decades of research demonstrating that the rhizosphere impacts bacterial-community structure (Grayston et al., 1998; Marilley et al., 1998; Smalla et al., 2001).

There is some debate as to whether antimicrobial secondary metabolites have a competitive function *in situ* (Hibbing et al., 2010). Recent studies have shown that secondary metabolites, previously recognized for their antibiotic activity, influence biofilm formation in *Bacillus subtilis* and may actually function in cell-cell communication (López et al., 2009). The development of biofilms at the plant-root surface is a well-documented phenomenon and raises questions about whether the enrichment of antimicrobial traits in this study truly reflects an increase in competitive interactions or is an artifact biofilm formation (Danhorn and Fuqua, 2007). We would argue that traits associated with biofilm formation are also competitive traits (Table 1) and differential abundance testing lends weight to the hypothesis that the rhizosphere selected for competitive life strategists: OTUs identified as Actinobacteria, Sphingomonadales and Sphingobacterales exhibited the clearest enrichment due to the presence of the rhizosphere and are generally considered to be competitive eco-types, whilst Acidobacteria order Gp6, which are predicted to be oligotrophic in nature, exhibited a clear decline (Fierer et al., 2007; Aislabie and Deslippe, 2013).

### Stress-tolerant traits do not increase with cadmium dose in the rhizosphere

Cadmium is a toxic metal ion that damages thiol groups in proteins (Nies, 2003), as such we predicted that Cd would increase the prevalence of traits related to cellular maintenance (i.e., stress tolerance traits). Alpha-diversity, beta-diversity and differential abundance analyses all indicated that the impact of Cd on the community was less severe than that of the rhizosphere, and only significantly altered community structure under the highest Cd dose (Figures 1, 2, 5).

Cd-treated bulk-soil communities followed CSR predictions with the 20 mg kg^−1^ Cd dose scoring the same as base-line soils and the 100 mg kg^−1^ Cd dose scoring higher on the S axis. However, the relationship between Cd and S-traits in the rhizosphere was less clear as 0 and 100 mg kg^−1^ rhizosphere treatments scored higher on the S axis, whilst the 20 mg kg^−1^ Cd treatment did not (Figure 3). The inconsistent impact of Cd upon rhizosphere S-traits might be due to the low impact of Cd on these communities. In particular, ANOSIMs highlighted that although Cd altered OTU abundance *and* taxonomic structure in bulk soils, in the rhizosphere Cd had no significant impact on taxonomic structure (Figure 2). These results suggest that the rhizosphere somewhat buffers the impact of Cd upon rhizosphere communities. Our findings are in accord with previous research that demonstrated a similar protective effect of pakchoi rhizospheres upon bacterial community structure in the presence of low Cd loads (0.7–3.5 mg kg^−1^) (Shentu et al., 2014).

### Ruderal traits reveal the impact of the rhizosphere and cadmium

Ruderal life strategists are predicted to prevail in disturbed environments that are not resource-limited (Grime and Pierce, 2012). Although no explicit disturbance was applied to these microcosms, we predicted that CSR trade-offs would be revealed as an increase in R functional traits in the absence of competition (the rhizosphere) and stress (Cd dose). Ruderal traits, as defined by Grime, are “those that assist populations to re-establish in circumstances of frequent and severe disturbance” (Grime and Pierce, 2012).

Base-line soils scored highest on the R axis, which is in accordance with CSR predictions (Figure 3). The addition of the highest dose of Cd decreased R scores, possibly reflecting a shift toward slower, stress-tolerant growth-strategies. Similarly, the presence of the rhizosphere also decreased R scores, which might reflect the redirection of resource investment into the production of secondary metabolites used in competition. We also observed a synergistic reduction in R scores when both selective pressures were present, possibly reflecting the combined redirection of resources from growth into the production of competition-related compounds and stress tolerance mechanisms (i.e., CS-life strategists). Our findings are in accord with previous research that have used growth potential (specifically C mineralization rates) to discriminate between microbial R/*k* strategists (i.e., copiotrophs and oligotrophs), the former of which are analogous to ruderals in the CSR theory (Fierer et al., 2007; Grime and Pierce, 2012). These observations suggest that metrics of growth potential may constitute an excellent axis for discriminating between R and C or S life strategists.

### Microbial competition is important for microbial-assisted phytoextraction

We observed that that competitive traits were enriched in the rhizosphere even in the presence of cadmium and that the rhizosphere environment appears to reduce the impact of Cd upon the community. In the context of heavy-metal phytoremediation, these results underline the importance of competitive resource monopolization (as opposed to heavy metal resistance) in the ability of an isolate to occupy and proliferate in a Cd-contaminated rhizosphere.

## Limitations

We used predictive metagenomic profiling to assess changes to functional traits between microcosms. The predictive nature of PICRUSt is a potential limitation as the software relies on taxonomic similarities between environmental 16S rRNA sequences and published metagenomes to make robust predictions (Langille et al., 2013). However, for environments with moderate amounts of reference genomes, including soils and the human gut, Langille et al. (2013) have demonstrated that PICRUSt can make accurate metagenome predictions. Additionally, predicted growth-related traits could discriminate between R and C/S life strategists in a similar fashion to which empirically measured growth-related traits can discriminate between copiotrophic and oligotrophic taxa (Fierer et al., 2007). This reinforces PICRUSt as a valid approach for investigating microbial community-traits and applying existing ecological classification schemes to bacterial communities.

Although we observed that functional traits followed many of the predictions made by CSR theory, we do not propose that the current classification strategy is perfect. For instance, we hypothesized that traits related to cell-wall integrity would constitute stress-tolerant traits (Table 1). However, initial classification attempts resulted in an inverse relationship between community S-scores and Cd dose (Data not shown). This unexpected relationship was resolved when close examination of differential abundance tests indicated that the major OTUs enriched in the presence of Cd (and the rhizosphere) included the Sphingomonadales and Sphingobacterales. Both of these Orders have a high prevalence of sphingolipids (SL) in their cell membranes in place of lipopolysaccharide (LPS), which is a relatively rare feature amongst bacteria (Nikaido, 2003). Other common soil microorganisms also exhibit non-canonical cell-walls such as the Planctomycetes, which have entirely proteinaceous cell-walls lacking peptidoglycan (Fuerst and Sagulenko, 2011). The unusual cell-wall structures of these abundant taxa confound attempts to interpret cell-wall related traits and highlight the importance of utilizing functional traits that are highly conserved across all taxa. Thus, although membrane and cell-wall modifications have been observed in stress-adapted microorganisms (Gounot, 1991; Da Silveira et al., 2003; Waranusantigul et al., 2011; Garba et al., 2016), these traits were excluded from the final S-allocation.

Additionally, we identified a suite of traits, related to foraging (chemotaxis, plasticity, motility), which could not be neatly assigned within the CSR framework although they clearly constitute an important resource investment (Table S4). Possibly, definitions of CSR life strategist need to be rethought in order to accommodate macro- and micro-community traits.

Our current method is also limited by the need for pair-wise comparisons to a reference soil. This strategy limits the scope for comparing CSR classifications across studies, as outcomes are impacted by the choice of reference community. The development of a quantitative method for measuring traits and identifying key predictor variables that best discriminate between CSR life strategies, similar to the methods described by Hodgson et al. (1999) for plant CSR classification, would facilitate the interpretation and comparison of microbial communities from disparate environments.

## Conclusions

This study showed that bacterial traits can be used to discriminate between Grimes CSR life strategies and traits identified as competitive, stress tolerant and ruderal broadly followed the predictions of the CSR theory. In this study the use of ecological theory has revealed that competitive interactions are enriched in the rhizosphere even in the presence of cadmium from which the rhizosphere environment may offer a protective effect. These findings highlight the importance of understanding competitive interactions for the further development of microbial-assisted phytoremediation. Moreover, the core concepts of CSR theory appear to be generalizable to microbial communities and may be applicable to understanding community assembly across a variety of environments. The strategy for applying CSR classifications to microbial communities requires refinement. In particular, future research using controlled microcosms is needed to disentangle complex trait-environmental relationships observed *in situ* and to test specific questions and hypothesis (Wood et al., 2017). Microcosm studies using defined gradients of resource availability (stress) and disturbance will be essential in confirming ecological assumptions about functional traits, such as antibiotic production being related to competition, and to identify traits that routinely respond to stress and disturbance in predictable ways.

## Author contributions

Experimental design was conceived by JW in consultation with CT and AF. Glass house experiments were conducted by JW. Soil DNA extraction was performed by JW. Preparation of meta-barcoded 16S rRNA libraries for sequencing on Illumina MiSeq were carried out by JW. The assembly and QC of 16S community profiles was performed by JW. Traditional and trait-based bioinformatic analyses and statistical testing of 16S community profiles were researched, developed and performed by JW. JW, CT, and AF contributed to interpretation of data. Manuscript was drafted by JW; and JW, CT, and AF contributed to the revision and copy-editing of the final manuscript.

### Conflict of interest statement

The authors declare that the research was conducted in the absence of any commercial or financial relationships that could be construed as a potential conflict of interest.
