# Supplementary material for: Competitive Traits Are More Important than Stress-Tolerance Traits in a Cadmium-Contaminated Rhizosphere: A Role for Trait Theory in Microbial Ecology
Source: Front Microbiol. 2018 Feb 12;9:121. doi: 10.3389/fmicb.2018.00121 (PMC5816036; doi:10.3389/fmicb.2018.00121)
Supplement: Supplementary file 1 [file Table1.docx]

Supplementary Material

**Competitive traits are more important than stress-tolerance traits in a cadmium-contaminated rhizosphere: a role for trait theory in microbial ecology**

**Jennifer L. Wood^1^, Caixian Tang^2^, Ashley E. Franks^1*^**

*** Correspondence:** Ashley Franks; [A.Franks@latrobe.edu.au](mailto:A.Franks@latrobe.edu.au)

**Supplementary Data: rational and justification of CSR trait allocations**

**Table S1** **Justification of traits identified as competitive:** Traits that constituted an investment in monopolizing local resources were classified as competitive. Two main modes of resource monopolization were theorized:

1. Resource monopolization via increased capacity to capture local resources. Including traits pertaining to high-affinity transporters or molecules that capture rate-limiting nutrients, such as iron mobilization by siderophores. Cell size and shape (Young, 2006) would be another trait related to this classification, but is beyond the scope of this metagenomic analysis.
2. Local resource monopolization via direct inhibition of neighbors. Including physical inhibition of growth through biofilm formation and the production of secondary metabolite with antibiotic capability – see below for further discussion on this trait allocation.

| Putative competitive trait | Justification |
| --- | --- |
| ABC transporters | ABC transporters have roles in nutrient acquisition and osmoregulation and so can be classified as C or R traits depending on the types on transporters enriched. Transporters that increase nutrient acquisition include: sulphate and phosphate transporters as well as zinc, iron, manganese transporters.  Transporters enriched in no-Cd rhizosphere: sulphate, putrescine, phospholipid, phosphate, microcin C, zinc/manganese/iron, lipoprotein  Transporters enriched in the rhizosphere with 20 mg Cd kg^-1^: sulphate, putrescine, phospholipid, phosphate, microcin C, zinc/manganese/iron, lipoprotein |
| Acarbose and validamycin biosynthesis | The validamycin complex is an antibiotic produced by Streptomyces hygroscopicus var. limoneus that has been shown to be effective against a number of fungal taxa (Uyeda et al., 1985; Robson et al., 1988; Singh et al., 2006) |
| Biosynthesis of siderophore group nonribosomal peptides | Because levels of free ferric iron (Fe^3+^) in biological systems are always extremely low, there is serious competition for iron (Neilands, 1995; Glick, 2003; Ma et al., 2011). To improve Fe^3+^ uptake, microorganisms produce siderophores which have a much higher affinity for Fe^3+^ than plant phytochelatins (Burd et al., 1998; Glick, 2003; Chu et al., 2010). |
| Carbapenem biosynthesis | Carbapenem antibiotics are members of the beta-lactam family of antibiotics, the most important class of antibiotics currently in clinical use. They are active against many important Gram-positive and Gram-negative pathogens(Coulthurst et al., 2005). |
| Clavulanic acid biosynthesis | Clavulanic acid is a major β-lactam antibiotic produced by Streptomyces clavuligerus and is active against a wide spectrum of Gram-positive and Gram-negative bacteria (Saudagar et al., 2008) |
| Polyketide sugar unit biosynthesis  Biosynthesis of type II polyketide backbone  Biosynthesis of type II polyketide products | The term polyketide defines a class of molecules which include compounds of different structures and exhibit antibacterial, antifungal and antitumor properties (Vining, 1985; Katz and Donadio, 1993) |
| Staurosporine biosynthesis | Staurosporine is a protein kinase inhibiting antibiotic with antifungal and antitumor properties originally isolated from *Streptomyces staurosporeus* (Castro et al., 2010; Li et al., 2014) |

**Table S2 Justification of traits identified as stress-tolerance traits:** Environmental stress creates physiological challenges that threaten microbial function or survival (Schimel et al., 2007). Microbes must acclimate to immediate stress by altering their allocation of resources from growth to survival pathways. Two main modes of stress-tolerance were hypothesized:

1. The prevention of damage to the cell via processes that maintain the internal and external integrity of the cell. Including osmoregulation strategies such as the accumulation of ‘compatible solutes’ which can be synthesized or imported (Gouffi and Blanco, 2000), and traits that govern cell-wall and membrane integrity such as membrane fluidity which can be affected by changes in fatty acid composition within membrane (Yoon et al., 2015).
2. Responsiveness and alleviation of damage via processes such as the production of free radicle scavengers, in response to oxidative stress and production of reactive oxygen species (ROS), and DNA repair mechanisms.

Traits marked (*) were excluded from the final analysis as per main article discussion.

| Putative stress-tolerance trait | Justification |
| --- | --- |
| Alanine, aspartate and glutamate metabolism*  D-glutamine and D-glutamate metabolism* | D-alanine and D-glutamate are basic components of bacterial peptidoglycan layer (Horcajo et al., 2012). They provide resistance to most known proteases and thus contribute to protecting cell wall integrity. d-Ala and d-Glu are the most common d-amino acids present in the bacterial cell wall. However, d-Asp has been reported in the peptidoglycan of Lactococcus and Enterococcus (Cava et al., 2011). |
| Glycosphingolipid biosynthesis - globo series* | Sphingolipid-containing membranes are a relatively rare feature amongst bacteria and observations from the literature suggest that SL-containing membranes have a reduced permeability to small charged molecules (Kato et al., 1995; Kawahara et al., 1999; Nikaido, 2003; Ana et al., 2011). |
| Lipopolysaccharide biosynthesis* | Lipopolysaccharide is the major component in the outer leaflet of outer membrane in most Gram-negative bacteria. It provides the structural integrity of the outer membrane and variation in LPS oligosaccharide can change the surface hydrophobicity of the whole cell which impacts permeability (Wang et al., 2015)(Leive, 1974). |
| Peptidoglycan biosynthesis* | As a major component of the bacterial cell wall, bacterial growth and survival depends on peptidoglycan. Peptidoglycan plasticity is crucial for many other processes such as sporulation/germination, virulence,  morphological transitions, adaptation to stress conditions (*i.e.*, stationary phase), or exposure to antibiotics (Horcajo et al., 2012). |
| ABC transporters | ABC transporters have roles in nutrient acquisition and osmoregulation and so can be classified as C or R depending on the types on transporters enriched. Transporters that assist cellular osmoregulation include: Transporters of disaccharide osmoprotectants such as: sucrose, maltose, cellobiose, gentobiose, turnaose, palatinose and trehalose (López et al., 2009)^-^(Xu et al., 1998). Transporters for other osmoprotectants: K^+^, glutamate, glutamine, glycin betaine, proline betaine proline, ecotine and choline (Kuske et al., 2002). Other transporters related to stress tolerance include transporters for reactive oxygen species scavenges such as glutathione (Chesney et al., 1996; Ferguson and Booth, 1998; Smirnova et al., 2001)  R100: thiamin, mannopine, 2-aminoethylphosphonate, glycine betain/proline, osmoprotectant, maltose/maltodextrin, raffinose/stachyose/melibiose, lactose/L-arabinose, sorbitol/mannitol, alpha-glucoside, oligogalacturonide, trehalose/maltose, N-acetylglucosamide, cellobiose, multiple sugar, L-arabinose, rhamnose, fructose, glutamine, glutamate, dipeptide/heme/alpha-aminolevulnic acid, glutathione, iron (II)/manganese, capsular polysaccharide, heme |
| Ascorbate and aldarate metabolism | Ascorbate is a free radicle scavenger that has been reported as upregulated in both plants and bacteria in response to Cd stress (Kosová et al., 2011; Shamim and Rehman, 2015). A module search of upregulated KOs strongly indicated that KOs were related to ascorbate biosynthesis. |
| Mismatch repair | The loss or damage of genetic information leads to serious consequences. The protection of DNA and thereby the maintenance of the genome is indispensable for all living organisms (Rao and Prasad, 2016). |
| Porphyrin and chlorophyll metabolism | Mn-porphyrins are potent catalysts of O_2_^−^ dismutation and may have protective roles as antioxidants (Miriyala et al., 2012). A module search of upregulated KOs strongly indicated that KOs were related to vitamin B12 biosynthesis, a known antioxidant. |
| Proteasome | The ability to degrade protein is a potential adaption to environmental stress. In bacteria, proteosomes have been associated with resistance to nitric oxide stress, copper homeostasis, survival during iron starvation and the recycling of amino acids for protein the synthesis of new proteins under limiting conditions (Becker and Darwin, 2017). |
| Thiamine metabolism | Thiamine (vitamin B1) is essential for the normal growth and development of all living organisms. It plays a crucial role in carbohydrate metabolism, NADPH and ATP biosynthesis and the production of nucleic acid pentoses, and has been implicated in DNA repair in bacteria, yeast and plants (Makarchikov et al., 2003).  Adenosine thiamine triphosphate has been reported in bacteria, fungi and plants under specific metabolic conditions (e.g. amino acid starvation), suggesting that it has a role as an environmental stress protectant (Rapala-Kozik, 2011)(Gigliobianco et al., 2008). |

**Table S3 Justification of traits identified as ruderal:** Ruderal traits, as defined by Grime, are “those that assist populations to re-establish in circumstances of frequent and severe disturbance”. Two suites of traits were identified as proxies for microbial growth rate (i.e. the speed at which an organism can repopulate):

1. Investment metabolic processes that limit growth such as oxidative phosphorylation, and carbon metabolism.
2. Investment in machinery that limit growth such as ribosomes which have been shown to be predictive of cellular growth rates (Carrera et al., 2011).

| Putative ruderal trait | Justification |
| --- | --- |
| 2-Oxocarboxylic acid metabolism  Carbon fixation pathways in prokaryotes  Citrate cycle (TCA cycle)  Oxidative phosphorylation | An increase in core metabolic process permits rapid C utilization and rapid growth. Functional pathways associated with core metabolic processes include: central C metabolism, respiration, the citric acid cycle which unifies carbohydrate, fat, and protein metabolism (Fuchs, 2011), and the metabolism of 2-oxocarboxylic acids (such as pyruvate and oxaloacetate) which are key intermediates in several metabolic pathways. |
| Ribosome | Cellular machinery such as ribosomes have been shown, in non-oligotrophic microorganisms, to be a limiting resource that predicts cell growth rates (Fegatella et al., 1998; Carrera et al., 2011) |
| Aminoacyl-tRNA biosynthesis | Aminoacyl-tRNAs function to associate amino acids to tRNA transcripts during protein production. Their regulation is tightly coupled to active cell division (Heinonen, 1970). A higher aminoacyl-tRNA biosynthesis potential corresponds with faster doubling time. |
| Biosynthesis of amino acids  Pyrimidine metabolism | Amino acids are the building blocks of proteins and their availability limits the activity of tRNA transferase in protein production. |
| One carbon pool by folate | Folates play a key role in one-carbon metabolism essential for the biosynthesis of purines, thymidylate and hence DNA replication (Leive, 1974; Assaraf, 2007) |

**Table S4 Justification of traits identified as foraging traits:**  We identified several traits related to foraging behaviors: chemotaxis, plasticity and motility. Foraging traits are recognized as important life strategies for microorganisms and they are central to many ecological question, particularly in marine environments. Whilst foraging traits draw many parallels with ruderal traits they do not satisfy the Grimesian definition of a ruderal traits which is “traits that facilitate rapid repopulation after a disturbance”, where disturbance is defined as “the partial or complete destruction of biological material”(Grime and Pierce, 2012).

| Putative foraging trait | Justification |
| --- | --- |
| Bacterial chemotaxis | Detection of resources by bacterial chemotaxis is paramount for survival in nutrient-patchy environments where resources rapidly appear and disappear. Chemotaxis has been associated with ocean surface communities where nutrient sources are constantly in flux (DeLong et al., 2006)(Smriga et al., 2016) |
| Flagellar assembly R | Flagellation and motility enable bacteria to move towards resources in a patchy environment. In soils, several studies have shown that bacteria cannot move over measurable distances unless the soil is at saturation or near saturation (Moens and Vanderleyden, 1996)^,^(Smriga et al., 2016). However, motility has been show to facilitate inter-root travel (Bashan and Holguin, 1995) |
| Biotin metabolism  cAMP signaling pathway | Communication of nutrient status to transcription provides organisms with mechanisms for responding rapidly to metabolic needs and is essential for response to fluctuating environmental cues. Biotin status regulates gene expression in a broad range of organisms and is well understood in bacteria (Beckett, 2009). Signaling pathways such as cAMP pathways are necessary for decoding extracellular information and stimulating cellular responses (Camilli and Bassler, 2006). |
| Homologous recombination | Homologous recombination, which is also used in horizontal gene transfer to exchange genetic material between different strains and species of bacteria, can increase the acquisition of adaptive traits and constitutes a genotypic plasticity that can enhance survival in a changing environment (Gogarten and Townsend, 2005) |
| RNA degradation | The turnover of bacterial RNA facilitates rapid responses to environmental changes via the control and regulation of gene expression (Grunberg-Manago, 1999) |

**Table S5 Justification of traits that were unclassified in this study:** A number of traits identified as enriched in this study were left unclassified for one of the following reasons:

1. Traits relating to environment-specific resource consumption were not classified as C, S or R traits as they reflect the community’s adaption to resource type rather than stress, disturbance or general resource availability.
2. Classifications were not ascribed for traits lacking sufficient literature to support a C, S or R classification.
3. Classifications were not attempted for traits that are not sufficiently documented or described in prokaryotic systems.
4. Traits that were revealed upon manual inspection of the data, to be present as a subset of other traits were not classified.

| Unclassified trait | Rational |
| --- | --- |
| Benzoate degradation  Caffeine metabolism  Degradation of aromatic  Ether lipid metabolism  Fatty acid degradation compounds  Geraniol degradation  Glycine, serine and threonine metabolism  Glyoxylate and dicarboxylate metabolism  Inositol phosphate metabolism  Limonene and pinene degradation  Linoleic acid metabolism  Phenylalanine metabolism  Steroid degradation  Tryptophan metabolism  Tyrosine metabolism  Valine, leucine and isoleucine degradation | Traits that were likely to reflect the metabolism of plant root exudates were not classified. Root exudate compounds reported to be metabolized by bacteria include:  Amino acids (Campbell et al., 1997) – in particular various aromatic acids, such as benzoate, which are breakdown products of lignin, a main structural polymer of plants (Parales and Harwood, 2002).  Carbohydrates including m-inositol  Carboxylic acids including dicarboxylic acids, such as oxalic acid, of which dicarboxylates are the conjugate bases(Campbell et al., 1997)  Fatty acids, long-chain aliphatic acids including linoleic acid (Campbell et al., 1997) and steroid compounds, which are widespread in nature with many bacteria capable of degrading them as a carbon source (Donova and Egorova, 2012).  Terpenoid compounds such as limonene, pinene and geraniol (Owen et al., 2007)^-^(Lin et al., 2007)  Other metabolites, often thought to be allelopathic, such as caffeine (Morita et al., 2011; Sugiyama et al., 2016) |
| Atrazine degradation  Caprolactam degradation  Chlorocyclohexane and chlorobenzene degradation  Dioxin degradation  Polycyclic aromatic hydrocarbon  degradation  Fluorobenzoate degradation  Styrene degradation  Xylene degradation | Many man-made compounds have similar structural properties to aromatic root-exudes and have been shown to be elicit chemotactic effects and be degraded by microorganisms (Parales and Harwood, 2002). Functions relating to the metabolism of these compounds were not classified as C,S or R and, like root exudates, were considered to reflect the community’s adaption to resource type. These compounds included:  Persistent herbicides such as atrazine (Liu and Parales, 2009)  Synthetic polymers with aromatic structures, which share many catabolic enzymatic pathways used to catabolize root exudate aromatics such as benzene. These include: caprolactam (Rajoo et al., 2013), chlorobenzene, fluorobenzoate, dioxins (Bunge et al., 2003), styrene (Hartmans et al., 1990; Park et al., 2005) and xylene(Carmona et al., 2009) |
| Methane metabolism  Sulfur metabolism | Methylotrophs are ubiquitous in nature and have been found essentially in every environment tested, including extreme environments (Chistoserdova and Lidstrom 2013). However, in most environments, they are typically present at low relative abundances (Dedysh et al. 2001; Sauter et al. 2012; Beck et al. 2013). |
| Pentose and glucuronate interconversions | Pentose and glucoronate interconversions link fermentation to oxidative phosphorylation and were not classified because they were thought to reflect the metabolic fate of root exudates. |
| alpha-Linolenic acid metabolism | NC – lacking sufficient literature; The abundance of alpha-linolenic acid (ALA) have been implicated in pH (Rani and Agrawal, 2008) and cold stress (Sakamoto et al., 1998) and may be related to modifications of membrane fluidity. However, ALA research primary focus is on its antibacterial activity (Desbois and Smith, 2010) |
| Arginine biosynthesis | NC – lacking sufficient literature |
| Galactose metabolism | NC – lacking sufficient literature |
| Glycosaminoglycan degradation | NC – lacking sufficient literature. Some bacteria produce capsules which contain glycosaminoglycans. These may protect the cell from environmental stresses (Huang et al., 2013), but most research is regarding pathogenicity (DeAngelis, 2002). |
| Other glycan degradation | NC – lacking sufficient literature. Glycan is a major component of cell-wall peptidoglycan. KOs indicate synthesis of N-glycan which may translate to the modification of glycan strands in the peptidoglycan layer and stress tolerance. However there is insufficient evidence to classify this function (Vollmer, 2008) |
| Phosphonate and phosphinate metabolism | NC – lacking sufficient literature |
| Sulfur relay system | NC – lacking sufficient literature |
| Central carbon metabolism in cancer | NC - not relevant in prokaryotic systems |
| Drug metabolism - cytochrome P450 | NC - not relevant in environmental setting |
| Drug metabolism - other enzymes | NC - not relevant in environmental setting |
| Phenylalanine, tyrosine and tryptophan biosynthesis | NC – lacking sufficient literature |
| KEGG modules in global maps only | NC- not relevant in prokaryotic systems |
| Lysosome | NC- not relevant in prokaryotic systems |
| Peroxisome | NC - not relevant in prokaryotic systems |
| PPAR signaling pathway | NC - not relevant in prokaryotic systems |
| Steroid biosynthesis | NC – subset of another trait; Only one KO hit |
| Synthesis and degradation of ketone bodies | NC – subset of another trait. Strong overlap of with KOs from butanoate metabolism pathway |

Ana, D., Na, C., Bielawski, J., Hannun, Y.A., and Kasper, D.L. (2011). Membrane sphingolipids as essential molecular signals for Bacteroides survival in the intestine. *Proc. Natl. Acad. Sci. U.S.A.* 108(SUPPL. 1)**,** 4666-4671. doi: 10.1073/pnas.1001501107.

Assaraf, Y.G. (2007). Molecular basis of antifolate resistance. *Cancer and Metastasis Reviews* 26(1)**,** 153-181. doi: 10.1007/s10555-007-9049-z.

Bashan, Y., and Holguin, G. (1995). Inter-root movement of Azospirillum brasilense and subsequent root colonization of crop and weed seedlings growing in soil. *Microbial Ecology* 29(3)**,** 269-281. doi: 10.1007/BF00164890.

Becker, S.H., and Darwin, K.H. (2017). Bacterial proteasomes: Mechanistic and functional insights. *Microbiology and Molecular Biology Reviews* 81(1). doi: 10.1128/MMBR.00036-16.

Beckett, D. (2009). Biotin sensing at the molecular level. *Journal of Nutrition* 139(1)**,** 167-170. doi: 10.3945/jn.108.095760.

Bunge, M., Adrian, L., Kraus, A., Opel, M., Lorenz, W.G., Andreesen, J.R., et al. (2003). Reductive dehalogenation of chlorinated dioxins by an anaerobic bacterium. *Nature* 421(6921)**,** 357-360. doi: 10.1038/nature01237.

Burd, G.I., Dixon, D.G., and Glick, B.R. (1998). A plant growth-promoting bacterium that decreases nickel toxicity in seedlings. *Appl. Environ. Microbiol.* 64(10)**,** 3663-3668.

Camilli, A., and Bassler, B.L. (2006). Bacterial small-molecule signaling pathways. *Science* 311(5764)**,** 1113-1116. doi: 10.1126/science.1121357.

Campbell, C.D., Grayston, S.J., and Hirst, D.J. (1997). Use of rhizosphere carbon sources in sole carbon source tests to discriminate soil microbial communities. *Journal of Microbiological Methods* 30(1)**,** 33-41. doi: 10.1016/S0167-7012(97)00041-9.

Carmona, M., Zamarro, M.T., Biázquez, B., Durante-Rodríguez, G., Juárez, J.F., Valderrama, J.A., et al. (2009). Anaerobic catabolism of aromatic compounds: A genetic and genomic view. *Microbiology and Molecular Biology Reviews* 73(1)**,** 71-133. doi: 10.1128/MMBR.00021-08.

Carrera, J., Rodrigo, G., Singh, V., Kirov, B., and Jaramillo, A. (2011). Empirical model and in vivo characterization of the bacterial response to synthetic gene expression show that ribosome allocation limits growth rate. *Biotechnology Journal* 6(7)**,** 773-783. doi: 10.1002/biot.201100084.

Castro, A., Lemos, C., Falcão, A., Fernandes, A.S., Louise Glass, N., and Videira, A. (2010). Rotenone enhances the antifungal properties of staurosporine. *Eukaryotic Cell* 9(6)**,** 906-914. doi: 10.1128/EC.00003-10.

Cava, F., Lam, H., De Pedro, M.A., and Waldor, M.K. (2011). Emerging knowledge of regulatory roles of d-amino acids in bacteria. *Cellular and Molecular Life Sciences* 68(5)**,** 817-831. doi: 10.1007/s00018-010-0571-8.

Chesney, J.A., Eaton, J.W., and Mahoney Jr, J.R. (1996). Bacterial glutathione: A sacrificial defense against chlorine compounds. *Journal of Bacteriology* 178(7)**,** 2131-2135.

Chu, B.C., Garcia-Herrero, A., Johanson, T.H., Krewulak, K.D., Lau, C.K., Peacock, R.S., et al. (2010). Siderophore uptake in bacteria and the battle for iron with the host; a bird's eye view. *BioMetals* 23(4)**,** 601-611. doi: 10.1007/s10534-010-9361-x.

Coulthurst, S.J., Barnard, A.M.L., and Salmond, G.P.C. (2005). Regulation and biosynthesis of carbapenem antibiotics in bacteria. *Nature Rev. Microbiol.* 3(4)**,** 295-306. doi: 10.1038/nrmicro1128.

DeAngelis, P.L. (2002). Evolution of glycosaminoglycans and their glycosyltransferases: Implications for the extracellular matrices of animals and the capsules of pathogenic bacteria. *Anatomical Record* 268(3)**,** 317-326. doi: 10.1002/ar.10163.

DeLong, E.F., Preston, C.M., Mincer, T., Rich, V., Hallam, S.J., Frigaard, N.U., et al. (2006). Community genomics among stratified microbial assemblages in the ocean's interior. *Science* 311(5760)**,** 496-503. doi: 10.1126/science.1120250.

Desbois, A.P., and Smith, V.J. (2010). Antibacterial free fatty acids: Activities, mechanisms of action and biotechnological potential. *Applied Microbiology and Biotechnology* 85(6)**,** 1629-1642. doi: 10.1007/s00253-009-2355-3.

Donova, M.V., and Egorova, O.V. (2012). Microbial steroid transformations: Current state and prospects. *Applied Microbiology and Biotechnology* 94(6)**,** 1423-1447. doi: 10.1007/s00253-012-4078-0.

Fegatella, F., Lim, J., Kjelleberg, S., and Cavicchioli, R. (1998). Implications of rRNA operon copy number and ribosome content in the marine oligotrophic ultramicrobacterium Sphingomonas sp. strain RB2256. *Appl. Environ. Microbiol.* 64(11)**,** 4433-4438.

Ferguson, G.P., and Booth, I.R. (1998). Importance of glutathione for growth and survival of Escherichia coli cells: Detoxification of methylglyoxal and maintenance of intracellular K+. *Journal of Bacteriology* 180(16)**,** 4314-4318.

Fuchs, G. (2011). "Alternative pathways of carbon dioxide fixation: Insights into the early evolution of life?", in: *Annu. Rev. Microbiol.*).

Gigliobianco, T., Lakaye, B., Makarchikov, A.F., Wins, P., and Bettendorff, L. (2008). Adenylate kinase-independent thiamine triphosphate accumulation under severe energy stress in Escherichia coli. *BMC Microbiology* 8. doi: 10.1186/1471-2180-8-16.

Glick, B.R. (2003). Phytoremediation: Synergistic use of plants and bacteria to clean up the environment. *Biotechnology Advances* 21(5)**,** 383-393.

Gogarten, J.P., and Townsend, J.P. (2005). Horizontal gene transfer, genome innovation and evolution. *Nature Rev. Microbiol.* 3(9)**,** 679-687. doi: 10.1038/nrmicro1204.

Gouffi, K., and Blanco, C. (2000). Is the accumulation of osmoprotectant the unique mechanism involved in bacterial osmoprotection? *International Journal of Food Microbiology* 55(1-3)**,** 171-174. doi: 10.1016/S0168-1605(00)00192-6.

Grime, J.P., and Pierce, S. (2012). *The Evolutionary Strategies that Shape Ecosystems.* Oxford: Wiley-Blackwell.

Grunberg-Manago, M. (1999). "Messenger RNA stability and its role in control of gene expression in bacteria and phages", in: *Annual Review of Genetics.*).

Hartmans, S., Van der Werf, M.J., and De Bont, J.A.M. (1990). Bacterial degradation of styrene involving a novel flavin adenine dinucleotide-dependent styrene monooxygenase. *Appl. Environ. Microbiol.* 56(5)**,** 1347-1351.

Heinonen, J. (1970). Aminoacyl-tRNA synthetase formation and cell division in bacteria. *FEBS Letters* 9(5)**,** 252-254. doi: 10.1016/0014-5793(70)80368-4.

Horcajo, P., De Pedro, M.A., and Cava, F. (2012). Peptidoglycan plasticity in bacteria: Stress-induced peptidoglycan editing by noncanonical D-amino acids. *Microb Drug Resist* 18(3)**,** 306-313. doi: 10.1089/mdr.2012.0009.

Huang, H., Li, X., Li, N., and Zhong, W. (2013). Effect of environmental stresses on heparosan biosynthesis by Escherichia coli K5. *Journal of Pure and Applied Microbiology* 7(4)**,** 3025-3029.

Kato, M., Muto, Y., Tanaka-Bandoh, K., Watanabe, K., and Ueno, K. (1995). Sphingolipid Composition in Bacteroides Species. *Anaerobe* 1(2)**,** 135-139. doi: 10.1006/anae.1995.1009.

Katz, L., and Donadio, S. (1993). Polyketide synthesis: Prospects for hybrid antibiotics. *Annu. Rev. Microbiol.* 47**,** 875-912.

Kawahara, K., Kuraishi, H., and Zähringer, U. (1999). Chemical structure and function of glycosphingolipids of Sphingomonas spp and their distribution among members of the α-4 subclass of Proteobacteria. *Journal of Industrial Microbiology & Biotechnology* 23(4-5)**,** 408-413.

Kosová, K., Vítámvás, P., Prášil, I.T., and Renaut, J. (2011). Plant proteome changes under abiotic stress - Contribution of proteomics studies to understanding plant stress response. *Journal of Proteomics* 74(8)**,** 1301-1322. doi: 10.1016/j.jprot.2011.02.006.

Kuske, C.R., Ticknor, L.O., Miller, M.E., Dunbar, J.M., Davis, J.A., Barns, S.M., et al. (2002). Comparison of soil bacterial communities in rhizospheres of three plant species and the interspaces in an arid grassland. *Appl. Environ. Microbiol.* 68(4)**,** 1854-1863.

Leive, L. (1974). "THE BARRIER FUNCTION OF THE GRAM‐NEGATIVE ENVELOPE", in: *Annals of the New York Academy of Sciences.*).

Li, X., Huang, P., Wang, Q., Xiao, L., Liu, M., Bolla, K., et al. (2014). Staurosporine from the endophytic Streptomyces sp. strain CNS-42 acts as a potential biocontrol agent and growth elicitor in cucumber. *Antonie van Leeuwenhoek, International Journal of General and Molecular Microbiology* 106(3)**,** 515-525. doi: 10.1007/s10482-014-0220-6.

Lin, C., Owen, S.M., and Peñuelas, J. (2007). Volatile organic compounds in the roots and rhizosphere of Pinus spp. *Soil. Biol. Biochem.* 39(4)**,** 951-960. doi: 10.1016/j.soilbio.2006.11.007.

Liu, X., and Parales, R.E. (2009). Bacterial chemotaxis to atrazine and related s-triazines. *Appl. Environ. Microbiol.* 75(17)**,** 5481-5488. doi: 10.1128/AEM.01030-09.

López, M., Tejera, N.A., and Lluch, C. (2009). Validamycin A improves the response of Medicago truncatula plants to salt stress by inducing trehalose accumulation in the root nodules. *Journal of Plant Physiology* 166(11)**,** 1218-1222. doi: 10.1016/j.jplph.2008.12.011.

Ma, Y., Prasad, M.N.V., Rajkumar, M., and Freitas, H. (2011). Plant growth promoting rhizobacteria and endophytes accelerate phytoremediation of metalliferous soils. *Biotechnology Advances* 29(2)**,** 248-258.

Makarchikov, A.F., Lakaye, B., Gulyai, I.E., Czerniecki, J., Coumans, B., Wins, P., et al. (2003). Thiamine triphosphate and thiamine triphosphatase activities: From bacteria to mammals. *Cellular and Molecular Life Sciences* 60(7)**,** 1477-1488. doi: 10.1007/s00018-003-3098-4.

Miriyala, S., Spasojevic, I., Tovmasyan, A., Salvemini, D., Vujaskovic, Z., St. Clair, D., et al. (2012). Manganese superoxide dismutase, MnSOD and its mimics. *Biochimica et Biophysica Acta - Molecular Basis of Disease* 1822(5)**,** 794-814. doi: 10.1016/j.bbadis.2011.12.002.

Moens, S., and Vanderleyden, J. (1996). Functions of bacterial flagella. *Critical Reviews in Microbiology* 22(2)**,** 67-100.

Morita, A., Yanagisawa, O., Maeda, S., Takatsu, S., and Ikka, T. (2011). Tea plant (Camellia sinensis L.) roots secrete oxalic acid and caffeine into medium containing aluminum. *Soil Science and Plant Nutrition* 57(6)**,** 796-802. doi: 10.1080/00380768.2011.629176.

Neilands, J.B. (1995). Siderophores: Structure and function of microbial iron transport compounds. *Journal of Biological Chemistry* 270(45)**,** 26723-26726.

Nikaido, H. (2003). Molecular Basis of Bacterial Outer Membrane Permeability Revisited. *Microbiology and Molecular Biology Reviews* 67(4)**,** 593-656. doi: 10.1128/MMBR.67.4.593-656.2003.

Owen, S.M., Clark, S., Pompe, M., and Semple, K.T. (2007). Biogenic volatile organic compounds as potential carbon sources for microbial communities in soil from the rhizosphere of Populus tremula. *FEMS Microbiology Letters* 268(1)**,** 34-39. doi: 10.1111/j.1574-6968.2006.00602.x.

Parales, R.E., and Harwood, C.S. (2002). Bacterial chemotaxis to pollutants and plant-derived aromatic molecules. *Curr Opin Microbiol* 5(3)**,** 266-273. doi: 10.1016/S1369-5274(02)00320-X.

Park, M.S., Han, J.H., Yoo, S.S., Lee, E.Y., Lee, S.G., and Park, S. (2005). Degradation of styrene by a new isolate Pseudomonas putida SN1. *Korean Journal of Chemical Engineering* 22(3)**,** 418-424.

Rajoo, S., Ahn, J.O., Lee, H.W., and Jung, J.K. (2013). Isolation and characterization of a novel ε-caprolactam-degrading microbe, Acinetobacter calcoaceticus, from industrial wastewater by chemostat-enrichment. *Biotechnology Letters* 35(12)**,** 2069-2072. doi: 10.1007/s10529-013-1307-2.

Rani, P.S., and Agrawal, R. (2008). Effect on cellular membrane fatty acids in the stressed cells of Leuconostoc mesenteroides: A native probiotic lactic acid bacteria. *Food Biotechnology* 22(1)**,** 47-63. doi: 10.1080/08905430701863977.

Rao, D.N., and Prasad, Y. (2016). DNA repair systems: Guardians of the genome. *Resonance* 21(10)**,** 925-936. doi: 10.1007/s12045-016-0401-x.

Rapala-Kozik, M. (2011). "Vitamin b<inf>1</inf> (thiamine). a cofactor for enzymes involved in the main metabolic pathways and an environmental stress protectant", in: *Advances in Botanical Research.*).

Robson, G.D., Kuhn, P.J., and Trinci, A.P. (1988). Effects of validamycin A on the morphology, growth and sporulation of Rhizoctonia cerealis, Fusarium culmorum and other fungi. *Journal of general microbiology* 134.

Sakamoto, T., Shen, G., Higashi, S., Murata, N., and Bryant, D.A. (1998). Alteration of low-temperature susceptibility of the cyanobacterium Synechococcus sp. PCC 7002 by genetic manipulation of membrane lipid unsaturation. *Archives of Microbiology* 169(1)**,** 20-28. doi: 10.1007/s002030050536.

Saudagar, P.S., Survase, S.A., and Singhal, R.S. (2008). Clavulanic acid: A review. *Biotechnology Advances* 26(4)**,** 335-351. doi: 10.1016/j.biotechadv.2008.03.002.

Schimel, J., Balser, T.C., and Wallenstein, M. (2007). Microbial stress-response physiology and its implications for ecosystem function. *Ecology* 88(6)**,** 1386-1394. doi: 10.1890/06-0219.

Shamim, S., and Rehman, A. (2015). Antioxidative enzyme profiling and biosorption ability of Cupriavidus metallidurans CH34 and Pseudomonas putida mt2 under cadmium stress. *Journal of Basic Microbiology* 55(3)**,** 374-381. doi: 10.1002/jobm.201300038.

Singh, D., Seo, M.J., Kwon, H.J., Rajkarnikar, A., Kim, K.R., Kim, S.O., et al. (2006). Genetic localization and heterologous expression of validamycin biosynthetic gene cluster isolated from Streptomyces hygroscopicus var. limoneus KCCM 11405 (IFO 12704). *Gene* 376(1-2)**,** 13-23. doi: 10.1016/j.gene.2005.12.035.

Smirnova, G.V., Krasnykh, T.A., and Oktyabrsky, O.N. (2001). Role of glutathione in the response of Escherichia coli to osmotic stress. *Biochemistry (Moscow)* 66(9)**,** 973-978. doi: 10.1023/A:1012361323992.

Smriga, S., Fernandez, V.I., Mitchell, J.G., and Stocker, R. (2016). Chemotaxis toward phytoplankton drives organic matter partitioning among marine bacteria. *Proceedings of the National Academy of Sciences of the United States of America* 113(6)**,** 1576-1581. doi: 10.1073/pnas.1512307113.

Sugiyama, A., Sano, C.M., Yazaki, K., and Sano, H. (2016). Caffeine fostering of mycoparasitic fungi against phytopathogens. *Plant Signaling and Behavior* 11(1). doi: 10.1080/15592324.2015.1113362.

Uyeda, M., Ikeda, A., Machimoto, T., and Shibata, M. (1985). Effect of validamycin on production of some enzymes in rhizoctonia solani. *Agricultural and Biological Chemistry* 49(12)**,** 3485-3491. doi: 10.1080/00021369.1985.10867287.

Vining, L.C. (1985). Antibiotic biosynthesis. *Biotechnology Advances* 3(2)**,** 171-194. doi: 10.1016/0734-9750(85)90290-3.

Vollmer, W. (2008). Structural variation in the glycan strands of bacterial peptidoglycan. *FEMS Microbiol. Rev.* 32(2)**,** 287-306. doi: 10.1111/j.1574-6976.2007.00088.x.

Wang, Z., Wang, J., Ren, G., Li, Y., and Wang, X. (2015). Influence of core oligosaccharide of lipopolysaccharide to outer membrane behavior of Escherichia coli. *Marine Drugs* 13(6)**,** 3325-3339. doi: 10.3390/md13063325.

Xu, X., Abo, M., Okubo, A., and Yamazaki, S. (1998). Trehalose as osmoprotectant in rhodobacter sphaeroides f. Sp. Denitrificans il106. *Bioscience, Biotechnology and Biochemistry* 62(2)**,** 334-337. doi: 10.1271/bbb.62.334.

Yoon, Y., Lee, H., Lee, S., Kim, S., and Choi, K.H. (2015). Membrane fluidity-related adaptive response mechanisms of foodborne bacterial pathogens under environmental stresses. *Food Research International* 72**,** 25-36. doi: 10.1016/j.foodres.2015.03.016.

Young, K.D. (2006). The selective value of bacterial shape. *Microbiology and Molecular Biology Reviews* 70(3)**,** 660-703. doi: 10.1128/MMBR.00001-06.
